# Universality of periodicity as revealed from interlayer-mediated cracks

**DOI:** 10.1038/srep43400

**Published:** 2017-03-02

**Authors:** Myung Rae Cho, Jong Hyun Jung, Min key Seo, Sung Un Cho, Young Duck Kim, Jae Hyun Lee, Yong Seung Kim, Pilkwang Kim, James Hone, Jisoon Ihm, Yun Daniel Park

**Affiliations:** 1 Department of Physics and Astronomy, Seoul National University, Seoul, 08826, South Korea; 2 Department of Mechanical Engineering, Columbia University, New York, New York 10027, USA; 3 Institute of Applied Physics (IAP), Seoul National University, Seoul, 08826, South Korea; 4 Department of Physics and Graphene Research Institute, Sejong University, Seoul 143-747, South Korea

## Abstract

A crack and its propagation is a challenging multiscale materials phenomenon of broad interest, from nanoscience to exogeology. Particularly in fracture mechanics, periodicities are of high scientific interest. However, a full understanding of this phenomenon across various physical scales is lacking. Here, we demonstrate periodic interlayer-mediated thin film crack propagation and discuss the governing conditions resulting in their periodicity as being universal. We show strong confinement of thin film cracks and arbitrary steering of their propagation by inserting a predefined thin interlayer, composed of either a polymer, metal, or even atomically thin graphene, between the substrate and the brittle thin film. The thin interlayer-mediated controllability arises from local modification of the effective mechanical properties of the crack medium. Numerical calculations incorporating basic fracture mechanics principles well model our experimental results. We believe that previous studies of periodic cracks in SiN films, self-de-bonding sol-gel films, and even drying colloidal films, along with this study, share the same physical origins but with differing physical boundary conditions. This finding provides a simple analogy for various periodic crack systems that exist in nature, not only for thin film cracks but also for cracks ranging in scale.

Because crack propagation generally leads to catastrophic failure, understanding its mechanisms and methods for its prevention is of the highest interest^1^. Although we still do not fully comprehend some fundamental aspects of this phenomenon, our current understanding has already enabled novel applications. These applications range from forensics, in which a crack pattern reveals much about both the crack-formation event and the material properties of the medium^2^, to nanopatterning, in which cracks with high aspect ratios and nanometre-sized widths can be applied as nanotemplates for nanowire growth^3^ and the nanofabrication of electronic^4^ and microfluidic devices^5^,^6^ and even high-precision bio-mimicking sensors^7^.

But in the fundamental level of this phenomenon, in spite of the tremendous effort to understand crack propagation and resulting pattern, still there is no simple underlying theory capable of predicting patterns and associated instabilities for general cases. Because, to fully describe a crack pattern, a detailed account of many variables, including but not limited to stress distribution, mechanical properties, material homogeneity, and defects, is required; such an undertaking is nontrivial. Periodicity in such complicated systems could simplify the problem and may provide important clues to the general understanding of crack pattern. Thus, studies of periodicity in crack patterns^8^,^9^,^10^,^11^,^12^,^13^,^14^,^15^,^16^,^17^, especially a robust method to control such patterns, are invaluable. Furthermore, reports of such rare but useful phenomenon range various physical scales from those found in a quenched glass^16^ to cracks on the surface of ice sheets of Europa^17^, which hint at the existence of dimensionless universality.

Among these periodic crack systems, thin film periodic cracks, which are observed in material systems employed by the semiconductor industry process, are of high technological importance^10^,^11^,^12^,^14^,^18^. A thin film crack is the fracturing of the top nano- to micrometre thick coating on a substrate, usually without accompanying total fracture of the substrate. Thin film systems generated in clean environments are ideal for minimising defects and achieving homogenous crack medium; thus, undesirable disorders of a crack path can be minimised. So attempts of controlled crack generation in thin films have pursued recently, which is advantageous in the aforementioned nanofabrication techniques and device applications. So far, various attempts to control crack patterns in the thin film have focused on modifying stress and defects in the thin film and have shown relative success in predetermining and confining crack paths but essentially only in one dimension^6^,^14^,^19^,^20^.

Here, we focus on extrinsically modifying the fracture toughness of crack medium via material inhomogeneity. If two dissimilar materials are introduced as the crack medium, the resulting violation of material homogeneity will modify crack formation and dynamics accordingly. Thus, it is reasonable to consider whether crack paths could be predetermined if we are able to engineer a scalable method of locally controlling fracture toughness. Such a scheme will require evident modification of fracture toughness with minute changes in other material components and parameters. Whether such local fracture toughness modification can steer crack propagation in an arbitrary manner has not been demonstrated, nor even contemplated.

In this study, we locally impose a difference in fracture toughness by inserting a patterned thin layer *a priori* in between brittle amorphous SiO_2_ and a substrate, thereby forming a predefined track to govern the crack propagation. We find that crack propagation can be robustly confined to predetermined paths, both linear and curved, enabling the patterning of complex and arbitrary shapes. The path shape within a confined track is oscillatory. Due to its resemblance to other wavy crack systems, we could consider our cracks as the thin film case of ‘brittle adhesive joint cracks’^8^,^9^ and model them accordingly via numerical simulation incorporating basic fracture mechanics principles, which results in good agreement with the experimental crack pattern.

Although we illustrate the phenomenon in a nanoscale thin film, our results and analysis sheds a new light on the origins of the periodicity. Namely, we find no dimensional scale uniqueness; thus, this work could provide a simple relationship between various periodic crack phenomena.

## Results

### Interlayer-mediated generation of periodic wavy cracks

**Figure 1:**
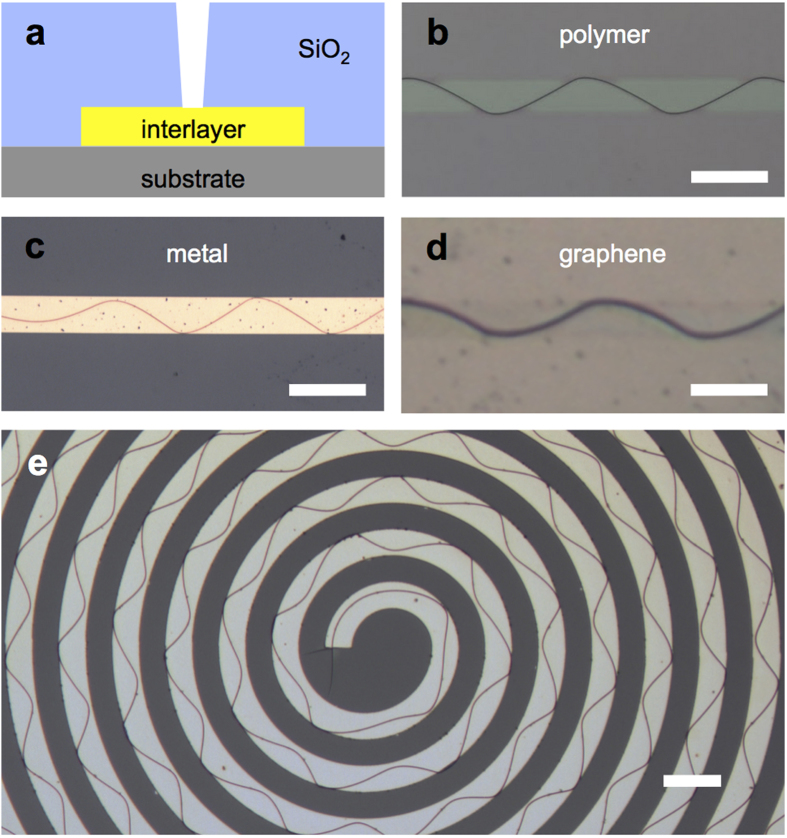
Interlayer-mediated wavy crack generation. (**a**) Cross-sectional schematics of the interlayer crack sample. (**b–e**) Optical micrographs of various interlayer samples in top view, (**b**) polymer, (**c**) metal, (**d**) graphene. (**e**) The spiral interlayer sample demonstrates the versatility of this method. All scale bars represent 40 μm.

**Figure 2:**
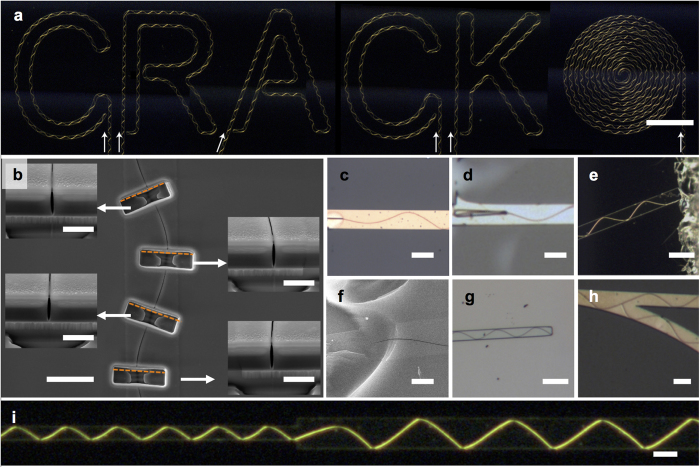
Writing complex shapes with controlled single continuous crack patterns and details of the crack pattern. (**a**) The word ‘CRACK’ and a spiral are written using cracks. Each crack begins from a scribed scratch at the lower section of the image and is guided through straight tracks to each letter-shaped track. White arrows indicate crack starting points and growth directions. The image was taken in the dark-field mode for better visibility. The scale bar represents 400 μm. (**b**) Cross-sectional scanning electron microscope (SEM) images of a metal interlayer crack sample. Cracks are perpendicular to the substrate without any phase-dependent tilting; no crack through the substrate was observed. Focused ion beam (FIB) milling was used to reveal cross-sections of different segments of the crack pattern. Before FIB milling, a 200-nm Al layer was deposited to reduce the charging effect during milling. The scale bar represents 10 μm; in insets, the scale bars represent 2 μm (**c**) A FIB-milled initiation notch. The scale bar represents 20 μm (**d**) Lithographically defined metal extrusion notch pattern, which is composed of 400-nm-thick Al. The scale bar represents 20 μm (**e**) Optical dark-field microscope image of the sample with scratch via manual scribing and (**f**) enlarged SEM image. The scale bars represent 40 μm and 5 μm respectively (**g**) Auto termination at interlayer track endpoint. The scale bar represents 40 μm. (**h**) Crack is stopped by another crack. The scale bar represents 20 μm. (**i**) Amplitude and period modulation in a single continuous crack with track width change. The scale bar represents 40 μm. All interlayer materials were 100-nm-thick Al, except (**c**), which was 100-nm-thick Cu.

Examples of ordered periodic crack patterns are shown in Fig. 1. A cross section schematic of a crack sample is shown in Fig. 1a. The crack medium is an ~2 μm thick SiO_2_ deposited on top of a substrate. In between the substrate and the brittle thin film, we inserted various interlayers, such as thin polymeric layers (Fig. 1b), metallic thin films (Fig. 1c) or few-layer graphene (Fig. 1d). Conventional lithography and deposition techniques were used to make these samples, and three different methods to initiate cracks were applied before or after the brittle medium deposition. After a thermal treatment, periodic cracks were observed on top of the inserted interlayer. The specific methods utilised to realise these crack samples are provided in the Methods section. We consistently observed single oscillatory-like crack patterns, which propagated without termination to the endpoints of a line-shaped (width: 20 μm) interlayer track, with lengths up to 3000 μm, or for a spiral track, with lengths up to 1.6 cm. We believe that there is no apparent reason for limiting the crack length as long as the interlayer track is continuous without a terminus. We also find the oscillatory pattern’s amplitude and periodicity to be dependent on track width (see Supplementary Materials Fig. S1). The spiral pattern clearly demonstrates the versatile manipulation of crack propagation; this study provides the first demonstration of truly two-dimensional patterning using a single continuous crack (Fig. 1e). With this maneuverability, we could even write complex patterns, such as letters (Fig. 2a).

Cross-sectional scanning electron microscope (SEM) images (Fig. 2b) show that the cracks are normal up to the substrate surfaces, with a typical gap at the SiO_2_ surface of 50–200 nm; no penetration through the interlayer or substrate in the vertical direction was observed. Thus, the cracks are confined to the SiO_2_ medium above the interlayer track. Also note that no phase-dependent tilting was observed, unlike previously reported high-stress silicon nitride cases^14^,^21^. The cracks could be initiated from either a focused ion beam (FIB)-milled notch (Fig. 2c) or a lithographically defined sharp protrusion (Fig. 2d), or even with random defects from hand scribing using cutting tools (Fig. 2e,f). Cracks terminated at the ends of the interlayer patterns (Fig. 2g) or when they met other crack patterns (Fig. 2h). Crack patterns respond immediately to the change of track width, so we are able to obtain amplitude and period modulation within a single continuous crack pattern (Fig. 2i). Finally, track width was positively correlated with the period of the crack pattern (Fig. S1). Compared with previous studies, this crack patterning is not limited by the crystallographic nature of the substrate^14^,^21^ or complexity of the notch array structures^6^.

### Role of the inserted interlayer

Notably, a similar result was recently reported when a metallic layer was inserted between a substrate and a SiN thin film^10^. The authors of that study attributed this phenomenon to the buckling of the film, which was induced by dewetting of the metallic film at high temperature. Although we observe a similar result with evaporated SiO_2_ and spin-on glass film (Fig. S2), unlike the previous study, no apparent buckling or dewetting was observed in the SEM cross-section images of the metallic interlayer samples. We investigate the crack occurrence with different interlayer materials and geometries, such as different thicknesses or widths of the interlayer. Periodic crack pattern occurrence is found to be highly dependent on the selected material rather than the thickness or width of the interlayer. For example, polymers such as polymethyl methacrylate (PMMA) and metals such as Au, Al, Cu, and Ni shows periodic crack generation, whereas Cr and Ti do not. When heated, thermal expansion of interlayer acts as a source of stress to the film. One could easily conclude that these results are because the thermal expansion of Cr and Ti is lower than those of other interlayer materials, which would result in lower thermal stress compared with the other interlayer materials. However, this crude simplification conflicts with the periodic cracks obtained with a graphene interlayer, because thermal expansion of graphene above room temperature is known to be negative or very small compared with conventional metals^22^.

**Figure 3:**
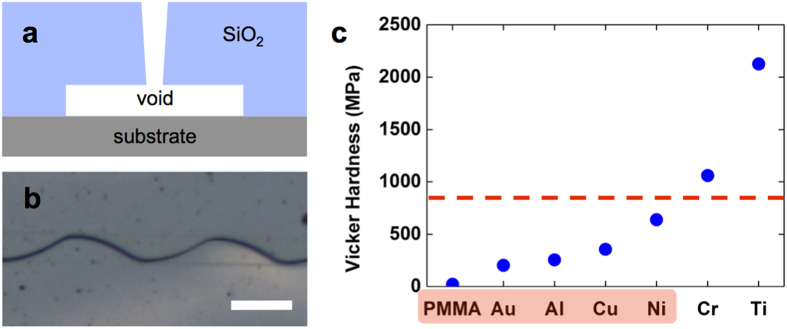
Void sample and role of the interlayer. (**a**) Cross-sectional schematics of the void sample. (**b**) Optical image of a void crack sample. The scale bar represents 40 μm. (**c**) Dependency of crack occurrence on interlayer hardness (Vickers). The red colour on material names indicates periodic crack generation.

To further investigate the role of the interlayers, we introduce a new sample scheme, referred to herein as the ‘void scheme’, which is demonstrated as a cross-sectional schematic in Fig. 3a, with its resultant pattern shown in Fig. 3b. We chemically etched out the metal interlayer before thermal treatment, leaving a tunnel-like void between the crack medium and substrate. If the role of the interlayer is to enhance or confine the stress on top of the interlayer region, then this elimination of the interlayer should hinder the generation of periodic cracks. However, our void samples still showed well-ordered similar periodic cracks. Based on the observation of the ‘void scheme,’ we hypothesise that the role of the interlayer is akin to a mechanical link, like a soft spring, between the substrate and crack medium. That is, the softness of the interlayer leads to effective fracture toughness reduction of the SiO_2_ on top of the interlayer compared with the SiO_2_ that directly adhered to the substrate. The fracture toughness of a thin film (with the substrate) depends not only on the toughness of the film’s material itself but also on the material properties of the substrate and adhesion strength between the film and substrate^23^. Thus, with help from the soft interlayer, the crack medium on top of the interlayer is much easier to be mechanically deformed and, in extreme cases, fractured^24^. In the void case, this effective softness is at the maximum level, which means that SiO_2_ above the void area has maximum freedom to be mechanically deformed. This compliance condition is supported by the fact that even with random scratch defects everywhere, cracks favour growing on top of the void (interlayer) region. Note that, in the case of ‘telephone cord delamination’, tailoring of delamination pattern via adhesion engineering was reported, which shares similar explanation we described above^25^.

Figure 3c shows the Vickers hardness plots of the interlayers that we tested; the results show that above a certain value of hardness (indicated by a red-dotted line in the graph), no periodic crack generation occur. In the graphene cases, due to its relatively low adhesion strength to the substrate or the crack medium, graphene acts as a void. After crack medium deposition, graphene inserted area, which is wider than 100 × 10 μm^2^, typically showed total delamination of SiO_2_ from the substrate (Fig. S3). This result clearly demonstrates the graphene samples’ weak adhesive nature.

**Figure 4:**
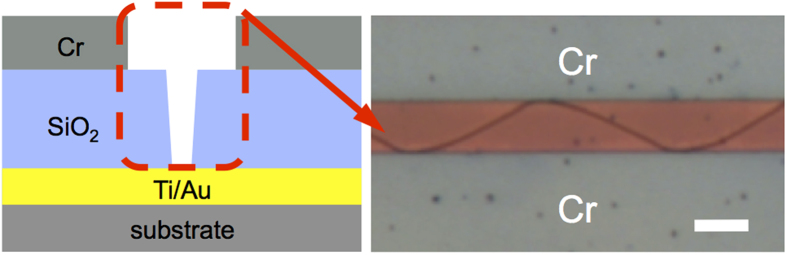
‘Negative reinforcement’ sample. (Left) Cut plane schematics of the sample. (Right) Optical image of its resultant crack pattern. The scale bar represents 20 μm.

To further test this hypothesis of effective fracture toughness modification, we devise another scheme, namely, ‘negative reinforcement’. The idea is that if we introduce a soft interlayer to the entire surface of the substrate, then the entire crack medium will have the freedom to form cracks. When we performed this experiment, a randomly oriented curvy network of crack patterns was observed everywhere (Fig. S4). However, when we added other top layers in parallel stripes shape, these top layers toughened the area covered and limit crack occurrence. So now cracks grow only within the area between these two additional stripes. This result shares same explanation with the simple line-shaped interlayer scheme, which we discussed in an earlier section. Figure 4 presents the cross-sectional schematics of this ‘negative reinforcement’ scheme and its resultant pattern, showing the expected periodic crack pattern confined within an area without an additional top Cr layer. This result shows that we could generate and control periodic crack patterns by modifying the fracture toughness of thin films.

### Crack path prediction from fracture mechanics

An important question still remains unanswered: why do cracks run in an oscillatory manner on top of the insertion layer rather than a random or straight manner? To gain insight regarding this wavy propagation, we identified similar phenomena in nature. We noted that one of the oldest examples, the so-called ‘brittle adhesive joints’, possesses similar physical features, such as (i) narrow line-like confinement of the crack medium and (ii) biaxial stress for a periodic crack pattern^8^,^9^. Based on this similarity, we suggest that one could perceive that our thin film crack is a 2D projection of a brittle adhesive joint crack. In the brittle adhesive crack case, a brittle medium (adhesive) is sandwiched between two ductile substrates (Al plate), which naturally leads to confinement of the brittle medium with the long line shape and symmetry over the axis orthogonal to the crack advancing direction. In our thin film crack case, the crack sample with a void could be simply perceived as a 2D slice of the brittle adhesive crack case if one agrees that suspended SiO_2_ is more compliant than other SiO_2_ that directly adhered to the substrate. Not all brittle adhesive cracks are oscillating, but in the case of biaxial loading, they are. In thermally expanding systems, such as that of our thin film cracks, this biaxial condition is naturally satisfied by the isotropic expanding nature.

There are reports of a numerical calculation of wavy ‘brittle adhesive joints’ based on the basic linear elastic fracture mechanics (LEFM) principle^26^; we assumed that as long as the physical mechanism is coherent, then replication of the periodic features of our thin film crack pattern with similar numerical calculations would occur.

With the maximum tangential stress (MTS) criterion, a crack will favour the path that maximises its local tangential stress at the crack tip. We rewrote the tangential stress component of the crack tip by taking account only of singular terms from the Williams expansion^27^, i.e.,


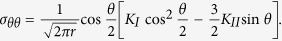


**Figure 5:**
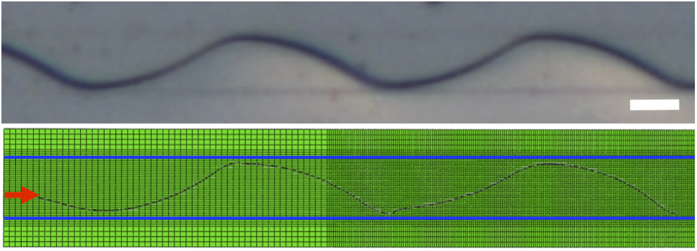
Numerical calculation of the crack path. (Top) Optical image of the void sample. The scale bar represents 20 μm. (Bottom) Calculated path of the crack. The red arrow indicates the crack initiation site.

Where *K*_*I*_ and *K*_*II*_ are the mode I and II stress intensity factors, respectively. By using the MTS criterion, we could calculate the angle of the newly generated crack tip segment. We used commercially available extended finite element methods (XFEM) software for the 3D modelling of our void crack sample and calculated the stress information at the crack tip. By repeating infinitesimal growth and recalculation of the angle, we could successfully simulate the crack path, which has a periodic wavy shape just as that of our experimental result (Fig. 5). For a more detailed procedure of the simulation, see the Methods section. The wavelength was approximately four times the width, in excellent agreement with the experimental results. The crack shape deviated from the exact sine function in the experiment (saw-tooth-like, asymmetry); this deviation was also observed in the simulation. This result is surprising because the stress component was simplified to singular terms (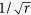), the fracture process was assumed to be linear, and no dynamics or time-dependent mechanism was applied. These speed-related dynamics are known to affect crack growth^28^. Although our crack growth speed was relatively fast (≫6 cm/s), our numerical calculation suggests that a quasi-static growth nature was dominant in our case.

### Crack growth by electron beam exposure

**Figure 6:**
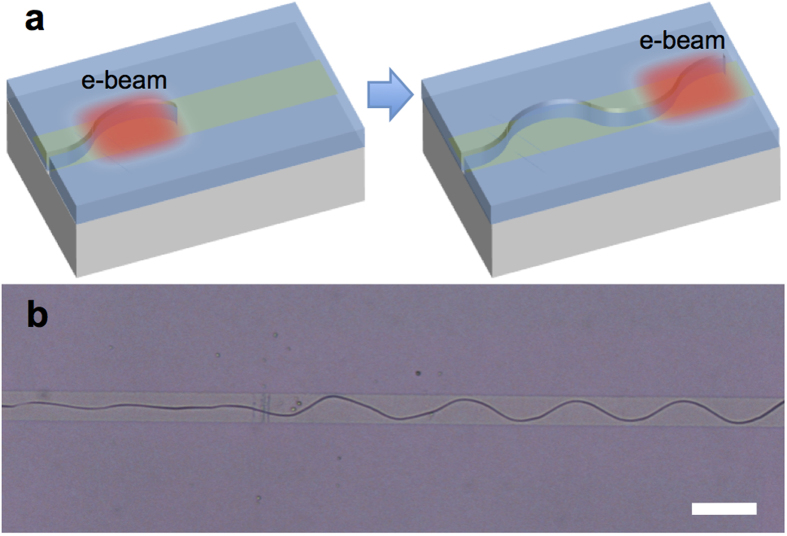
E-beam-induced crack growth. (**a**) Top view schematics of e-beam exposure procedure. (**b**) Optical image of the resultant crack pattern. The scale bar represents 40 μm.

Although our numerical calculation predicts periodic crack patterns well, one can question whether such quasi-static modelling best describes the actual crack formation observed. So here we demonstrate an experiment condition that is best analogous to the aforementioned simulation. We generate a periodic crack pattern by local electron beam exposures. Figure 6a provides a schematic procedure for crack generation via e-beam exposure. The red area indicates the e-beam exposed area. Due to the size of the field of view of the SEM, e-beam exposure was limited to 100 × 100 μm^2^, and a crack started to grow from the FIB-milled initiation notch while the surface of SiO_2_ was being scanned with 1 nA at 20 keV acceleration voltage. This crack stopped growing when it reached the outside of the scanned region and started to regrow when the uncracked region was exposed to the e-beam; thus, this crack growth is basically a ‘stop and go’ process. Figure 6b shows an optical micrograph of the resultant crack pattern on PMMA interlayer sample; a real-time record of this crack generation can be found in the Supplementary Material (Movie S1).

With e-beam irradiation, we could generate several possible sources of stress, such as heating and electrostatic charging^29^. If we consider heating from an e-beam as a source of stress, we expect the temperature to rise to ~ 300 °C, which is the lowest temperature for PMMA sample cracking with thermal treatment. However, even with overestimated heating due to e-beam irradiation, the expected temperature change is ΔT = ~2 K (Supplementary section 2–1 for detailed estimation). This result is also consistent with earlier reports of 70 K for SiO_2_ film on a Si substrate with a much higher current (600 nA) level and much smaller exposed area (2 × 2 μm^2^)^30^. On the other hand, if electrostatic charging could induce an equivalent level of stress compared to that of thermal stress at 300 °C (which is approximately 50 MPa, Supplementary section 2–2), then the charging effect could be the source of crack growth. Assuming that the FIB-milled initiation notch gap structure is a simple parallel capacitor plate with a 200 nm gap, then ~650 V surface potential is sufficient for crack growth (Supplementary section 2–3). With high energy, as in this study (20 keV), and a relatively thick coating of 2 μm, it is possible for the surface potential of the SiO_2_ layer to reach the kV level^31^. Additionally, we could not eliminate the possibility of plastic compaction by irradiation energy from an electron, which could reach the 100 MPa stress level in some cases^32^.

Although the specific mechanism of these electron beam stimuli is unclear, this example implies the following two important characteristics of this periodic crack system: (i) the periodic nature is dominated by geometry rather than dynamics, and (ii) the periodic nature is governed by local physical parameters because this crack was generated by local stimuli rather than a globally subjected thermal stress. Thus, we suggest that this result supports our quasi-static numerical calculation. This example also demonstrates a new way to control crack growth, which deserves further study.

### Applications of interlayer-mediated crack

**Figure 7:**
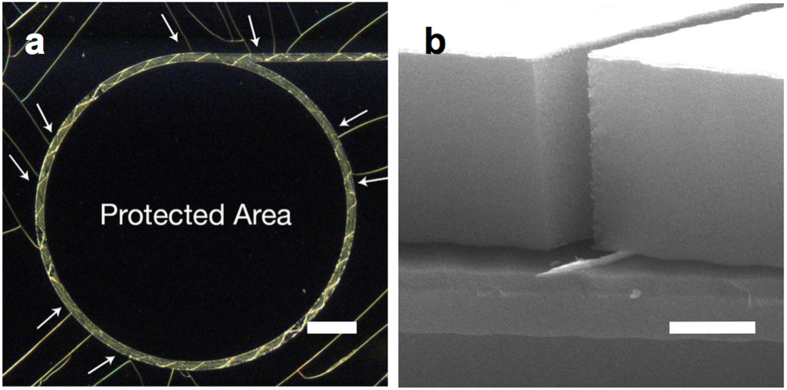
Applications of crack. (**a**) Optical dark-field image of demonstration of crack protection by using closed loop crack pattern. Outer random cracks (indicated by white arrows) are stopped by pre-existing closed loop crack. The scale bar represents 100 μm. (**b**) Cross-sectional SEM image of a metal nanowire template demonstration. Pt 100 nm was evaporated through the crack gap. Scale bar represents 1 μm.

Application-wise, such as other crack patterning studies, this crack pattern could be used as a nanowire generation template (Fig. 7a) and nanofluidic application (Movie S2). Because this method can generate arbitrary shapes with single continuous cracks and interlayer can be strategically removed, it is practically more favourable compared with the studies of controlled cracks accompanying substrate cracks^14^,^19^.

By exploiting the truly two-dimensional control of cracks, we could demonstrate material toughening with cracking (Fig. 7b). A closed loop-shaped single crack will protect inner regions from trespassing cracks from the outside. We have already demonstrated that transparent materials such as graphene and polymer can be used as interlayers. Thus, this technique could be an interesting protection method for display applications.

## Discussion

To understand the oscillating pattern of crack formation in this study, we have considered the possible deflection of the crack in an inhomogeneous interface. Previously, we described that the fracture toughness of SiO_2_ is extrinsically modified by the existence of an interlayer underneath; this same analogy can also be applied to the effective elastic stiffness of SiO_2_. Cracks remain only on the top SiO_2_ layer; thus, this crack system can be effectively simplified as a two-dimensional system with inhomogeneous fracture toughness and elastic stiffness over a surface plane. Then, the side edges of the interlayer region can be considered as the interface between two dissimilar materials. A crack obliquely approaching an interface, where the material over the interface is stiffer than where the crack resides, tends to curve away from the interface to increase the energy release rate^33^. The increase in the energy release rate approximately coincides with the increase in MTS, which is used in our numerical calculation. So the edge of the interlayer, the interface, changes its role with respect to each growth sequence of a crack. Such phenomenon has also been observed in a study of the dynamics of a liquid droplet^34^. A crack tip under biaxial stress will veer away from its original direction, which means a straight crack is unstable under this condition^35^. Thermal stress in our case is not uniaxial; thus, any crack running in the interlayer region is easily destabilised, such as the role of residual stress in ‘brittle adhesive joints’ cases. Ben Freund suggested a similar qualitative view for a nanoscale grating experiment^36^, but there is still no general theory that provides a clear and simple picture of how this periodic crack path is selected. In our experiment, we indeed observed amplification and saturation of the wavy crack amplitude, which supports those qualitative explanations (Fig. S5). We could simply summarise the necessary conditions for our confined oscillating cracks as material inhomogeneity, which results an effective fracture toughness modification and biaxial stress.

We believe that this type of necessity for a periodic crack can also be applied to other periodic cracks with different geometries and scales as demonstrated in Table 1. Because in our numerical calculation, there were no parameters or boundary conditions applicable only in nanoscale. As long as the crack medium thickness is relatively thin enough compared with the length scale of total structures, one can envision that the void scheme analogues in much larger systems could predict similar periodicity. So we compare the ratios between the thickness of the crack medium and the period of the crack patterns in various periodic crack systems (Table 1). Including this study, most of the periodic cracks in nature satisfy this ratio to be less than 0.1. This fact supports the universality of our modelling.

First, most periodic cracks in thin dielectric films are accompanied by self-local debonding^11^,^12^, which could be considered as a similar case to the void sample in our study. In these works, the self-debonding process generates fracture toughness modification around the crack tip as it grows, whereas the shrinking nature of the sol-gel films yields biaxial stress. Similar crack patterns are also observed with local debonding in Mo/Si multi layers for X-ray optics application^37^. Wavy cracks are observed in drying colloidal film. In this study, first, parallel straight cracks occur, and wavy cracks grow inside of them. Stress near these straight cracks will be relieved; thus, new cracks will veer away from the side boundaries to maximise released energy^13^.

Crack as a self-boundary concept can be applied to much larger cylindrical geometry brittle medium systems, e.g., a pressurised glass tube and industrial pipe^38^,^39^. In these cases, wavy cracks themselves are self-limiting boundaries, as straight cracks in drying colloidal cases and pressure from the inside introduces biaxial stress. We could find similarity in other interesting cases, such as tearing industrial wrappers^40^,^41^. Inhomogeneity will be met by film adherence to the frame at both sides, and blunt tools will introduce the biaxial stress condition to the system. We want to emphasise that the above examples and our study share an exotic similarity with respect to crack patterns, which is a correlation between the saw-tooth-like shape and crack growth direction (Fig. S1). This striking similarity between various periodic cracks in different scales has never been reported.

In nature, most crack media experience biaxial stress due to either expansion or contraction, which means that pure uniaxial stress is rare. Thus, we could suggest that when a wavy crack occurs, there might be a crack confinement condition, such as that found in this study, hidden inside. As mentioned before, although this study is based on thin film systems, the underlying mechanism is dimensionless. Thus, it might yield new insight for understanding different periodic crack systems, even for much bigger and untestable phenomena such as cracks in Earth’s crust or cracks in the ice of Europa^17^.

In summary, we demonstrated an easy and versatile method to control oscillating crack propagation in thin films using interlayers. We showed a truly two-dimensional steering of single continuous cracks in an arbitrary way and also demonstrate e-beam exposure-induced crack growth. From our experimental results and simulation, we suggest that modification of the fracture toughness by using an interlayer and biaxial stress can generate this peculiar periodicity. As we find no dimensional uniqueness in our model, this study yields fresh insight, which could relate other periodic crack systems in nature with varying scale. This technique could also open up new possibilities in micro and nanopatterning fields and provide a versatile on-chip platform for the dynamic fracture research area.

## Methods

### Sample fabrication

To define the metal interlayer pattern on top of the substrate, [SiO_2_ (300 nm)/Si (500 μm) or Si] conventional e-beam lithography was used. Using the PMMA pattern as a lift-off mask, 50–300-nm thick metal thin films were evaporated. Typically, 100 nm was used. To ensure good adhesion, brief oxygen plasma cleaning was pursued before metal evaporation. After the metal interlayer had been defined on top of the substrate, another oxygen plasma cleaning step was performed prior to SiO_2_ evaporation. To define the graphene interlayer, first, a large area (few cm^2^) of few-layer graphene was transferred to the substrate. Graphene was grown via Plasma Enhanced Chemical Vapor Deposition. The PMMA pattern was defined on top of the graphene via conventional e-beam lithography. Graphene was etched via oxygen plasma using PMMA as the etch mask; the remaining PMMA on top was removed by an organic remover such as Acetone. Polymer interlayer was defined by using a simple lithography step without any further process.

To make the initial defect, simple scratching was inflicted on the substrate surface across the interlayer pattern using a scribe tool, such as a diamond scribe. This process generates randomly oriented defects on top of the sample. FIB milling was also used to define nanoscale trench defects, and for some metal interlayer samples, metallic notch protrusion was additionally added on top of the interlayer to generate 3D notch-like defects in a subsequent SiO_2_ deposition. These scratches, trenches and notch protrusions act as crack initiators in the final annealing process.

We deposited SiO_2_ (99.999%, fused), with a thickness of ~2 μm, on the sample surfaces using e-beam evaporation. The annealing process was performed in a conventional tube furnace system (or annealing vacuum chamber) with a dry nitrogen flow (or vacuum). The rate of heating (<10 K/s) was controlled to yield the optimal results. Typically, crack generation occurred from 300–550 °C, depending on the interlayer materials.

### Void sample fabrication

Starting from 100-nm-thick Al (or Cu) metal interlayer samples, we hand scribed using a diamond scribe after SiO_2_ deposition and immersed the sample in diluted hydrochloric acid solution (or ferric chloride-based Cu etchant). By capillary force the etchant travelled underneath SiO_2_ through the scratched incision and dissolved the metal layer, leaving void-like tunnel structures. Samples were rinsed using clean DI water and Isopropanol consecutively, followed by N_2_ drying.

### ‘Negative reinforcement’ sample fabrication

First, the Ti/Au 5/50 nm bilayer was evaporated on the whole surface of the substrate. Then, 2-μm-thick SiO_2_ was evaporated. Finally, a 200-nm-thick Cr pattern was defined by conventional e-beam lithography and metallization.

### E-beam exposure-induced crack growth

Starting with a 70-nm-thick PMMA interlayer sample, we made a 10 μm × 2 μm × 200 nm (length × depth × width) trench using the FIB milling process. Our machine is also equipped with *in situ* SEM; thus, after successful notch fabrication, we scanned the notch pattern with a 1 nA current, 20 kV acceleration, 3 s per one scan, 100 × 100 μm^2^ view area. When a crack started to grow from the notch pattern, the scanning area was moved, following the crack tip growth.

### Numerical calculation of crack path

We chose the void scheme for calculation due to its simplicity. To predict the crack propagation path, the finite element method implemented in Abaqus/Standard 6.12 was used. In this case, the finite element analysis was linear. The crack growth simulation was based on and modified from previous studies^26^,^42^. The 3D crack was modelled by using an extended finite element method (XFEM) to avoid time-consuming re-meshing^43^. The element type was iso-parametric linear 3D elements with reduced integration (C3D8R), as provided by the commercial Abaqus option. The initial crack tip was introduced as a simple discontinuation plane, which is orthogonal to the substrate plane. The actual crack growth procedure was not performed using the 3D growth feature in Abaqus; instead, the pseudo 2D propagation iteration procedure was used, as in previous studies^26^,^42^. The crack growth direction (angle) was determined by using the maximum tangential stress (MTS) criterion^27^. To calculate this angle, stress intensity factor values from the interaction integral^44^ method of the program were used. This feature is implemented in the stationary crack calculation of Abaqus. Because the crack growth angle is given, crack growth is analogous to solving a first-order differential equation, 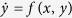, where the crack grows along the *x*-axis (initial crack tip direction), *y(x*) is the deviation of the crack from *x*-axis, and *f(x, y*) is the tangent of the crack growth angle. This differential equation was solved by using the improved Euler’s method for better accuracy^45^.

The detailed condition for the finite element modelling is as follows. The substrate’s Young’s modulus, Poisson ratio, and coefficient of thermal expansion (CTE) are 150 GPa, 0.22 and 3.4 × 10^−6^ K^−1^, respectively. The SiO_2_ film’s properties are 72.7 GPa, 0.16 and 0.5 × 10^−6^ K^−1^. In the simulation, the substrate’s CTE was effectively set to zero by accounting only for the difference of CTE for simplification. The 3D geometry of the model is illustrated in Fig. S6. The initial crack tip length was 10 μm. The size of the model and mesh were carefully tested and chosen to obtain a practical and saturated stress value around the crack tip. The thickness of the SiO_2_ film was 2 μm and the thickness of the Si substrate was reduced to 2 μm for fast calculation. The height and width of the void were 0.1 and 20 μm. The most coarse mesh size was 0.7 × 0.7 × 0.1 μm^3^ (xyz). Thermal loading was applied by increasing the model temperature up to 500 °C. At this elevated temperature condition, it was assumed that fast fracture occurs as in the experiment and the crack propagates. The crack propagation direction was calculated by using the MTS criterion, which uses stress intensity factors evaluated from the top surface of the film. The discrepancy of the crack propagation angle through the depth of the SiO_2_ film is marginal, being less than 10° difference for extreme cases. This in-depth variation was calculated from the finite element model, with conventional wedge elements of 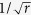 singularity, instead of XFEM to obtain more accurate values.

## Additional Information

**How to cite this article**: Rae Cho, M. *et al*. Universality of periodicity as revealed from interlayer-mediated cracks. *Sci. Rep.* **7**, 43400; doi: 10.1038/srep43400 (2017).

**Publisher's note:** Springer Nature remains neutral with regard to jurisdictional claims in published maps and institutional affiliations.

## Supplementary Material

Supplementary Information

Supplementary Movie 1

Supplementary Movie 2

## Figures and Tables

**Table 1:** Various periodic cracks in nature.

Periodicity scale	Medium thickness/period	Medium	Crack confinement	Biaxial stress	Ref.
μm	0.006–0.1	SiO_2_	Interlayer	Thermal stress	This study
μm	0.005–0.05	Sol-gel dielectric	Self-debonding	Drying densification	11, 12
μm	0.007–0.009	Mo/Si superlattice	Self-debonding	Thermal stress	37
μm	0.014–0.13	Colloidal latex or silica	Straight cracks nearby	Drying densification	13
cm	0.015–0.06	Glass	Cylindrical shape	Hydraulic pressure	38
m	0.03–0.1	Polyethylene	Cylindrical shape	Hydraulic pressure	39
mm	0.001–0.006	Polypropylene	Attachment to side frames	Blunt tool	40, 41
km	0.03	Ice	?	Water freezing	17
