# Supplementary material for: Universality of periodicity as revealed from interlayer-mediated cracks
Source: Sci Rep. 2017 Mar 2;7:43400. doi: 10.1038/srep43400 (PMC5333109; doi:10.1038/srep43400)
Supplement: Supplementary Information [file srep43400-s1.pdf]

Supplementary information

Title: Universality of periodicity as revealed from interlayer-mediated cracks

Myung Rae Cho[1], Jong Hyun Jung[1], Min key Seo[1,a], Sung Un Cho[1], Young Duck Kim[2], Jae Hyun Lee[1,3], Yong Seung Kim[4,b], Pilkwang Kim[1], James Hone[2], Jisoon Ihm[1,c] and Yun Daniel Park[1,3,*]

Author affiliations:
[1]Department of Physics and Astronomy, Seoul National University, Seoul, 08826, South Korea.
[2]Department of Mechanical Engineering, Columbia University, New York, New York 10027, USA.
[3]Institute of Applied Physics (IAP), Seoul National University, Seoul, 08826, South Korea
[4]Department of Physics and Graphene Research Institute, Sejong University, Seoul 143-747, South Korea.
[a]Present affiliation: Nanoelectronics Group, Service de Physique de l'Etat Condensé, CEA Saclay, CEA Saclay 91191 Gif-sur-Yvette cedex, France.
[b]Present affiliation: Samsung Electronics, Gyeonggi-do 446-711, South Korea.
[c]Present affiliation: Department of Physics, Pohang University of Science and Technology, Pohang 37673, South Korea.

Corresponding author:
[*]Yun Daniel Park, Department of Physics and Astronomy, Seoul National University, Seoul, 151-747, South Korea, +82-2-885-2361, parkyd@phya.snu.ac.kr

Section 1. Supplementary figures

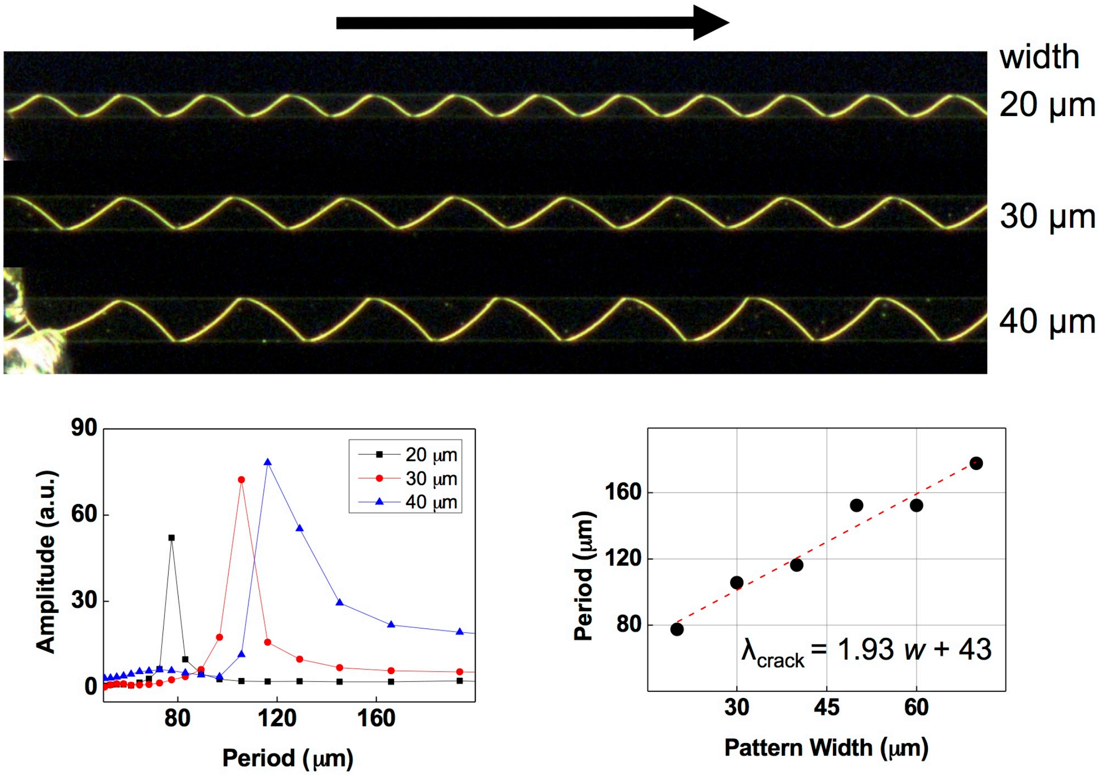

Fig. S1. Period dependency on interlayer track width. Black arrow on top indicates crack growth direction. The bottom left graph is the Fourier coefficient of the digitized crack patterns from optical images. Single dominant peaks were observed for each case, with positive correlation with track width, as indicated in the bottom right graph. Note that saw-tooth-like features in the crack pattern are more pronounced in the wider track width sample. Additionally, the crack running direction and saw-tooth-like features have a strong correlation; no reversed case was observed.

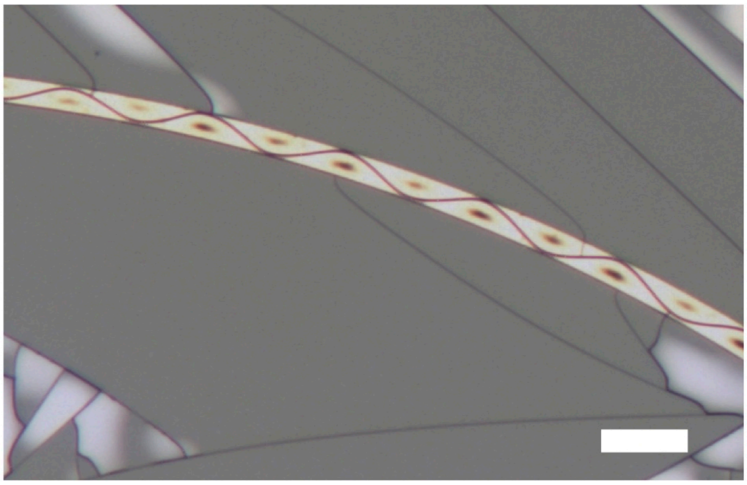

Fig. S2. Hydrogen silsesquioxane (HSQ)-coated sample. Conventional spin on glass material HSQ was used instead of evaporated SiO_2. The interlayer material was Al 100 nm. A crack pattern similar to that of the evaporated SiO_2 sample was observed. Scale bar represents 20 μm.

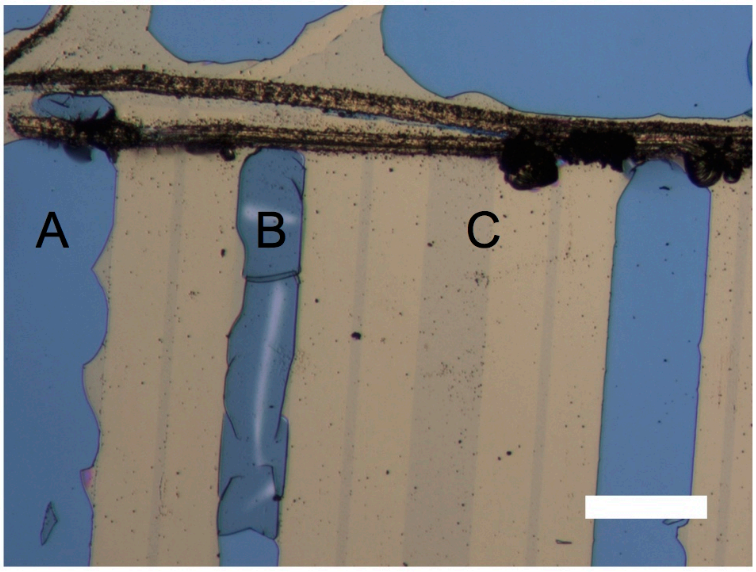

Fig. S3. Delamination of SiO_2 after evaporation for a graphene interlayer sample. Blue area (A) indicates exposed substrate surface due to SiO_2 delamination and khaki color area indicates SiO_2 covered area. Due to the presence of the graphene interlayer, parts of the SiO_2 covered area are darker than other regions. In B, partial delamination and buckling of the film are shown. Scale bar represents 200 μm.

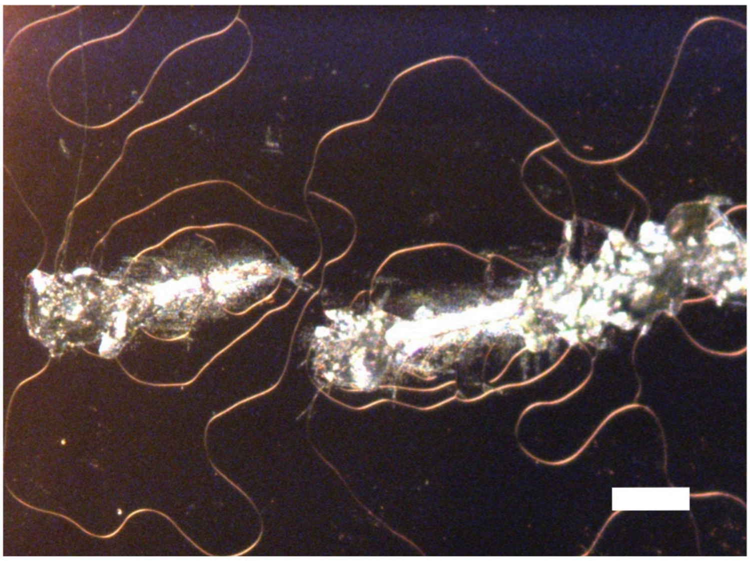

Fig. S4. A worm-like network of crack generation in the case of the entire substrate being covered with a metal interlayer. The metal interlayer was Cu 100 nm and SiO_2 2 μm was evaporated on top of the Cu layer. This image is a dark-field optical image; scale bar represents 200 μm.

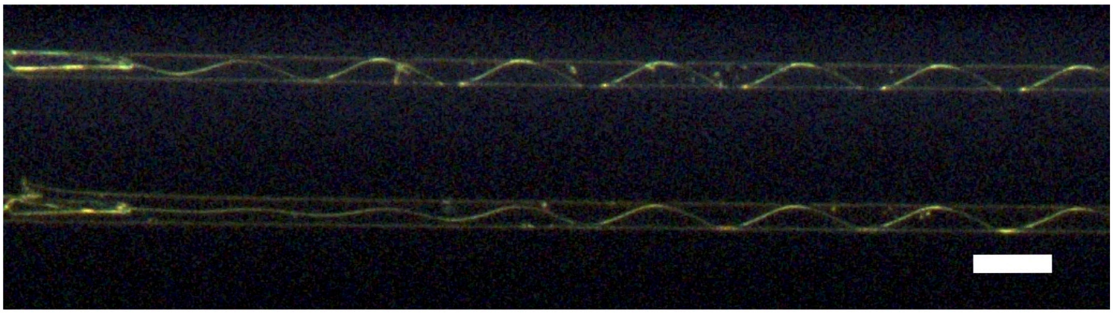

Fig. S5. Dark-field image of Al interlayer sample, showing amplification of the waveform. Cracks are started from the initiation notches in the left corner. Although two crack patterns have a similar final period, the bottom one starts from a small amplitude and soon saturates to its usual waveform. Track width is 15 μm and scale bar represents 40 μm.

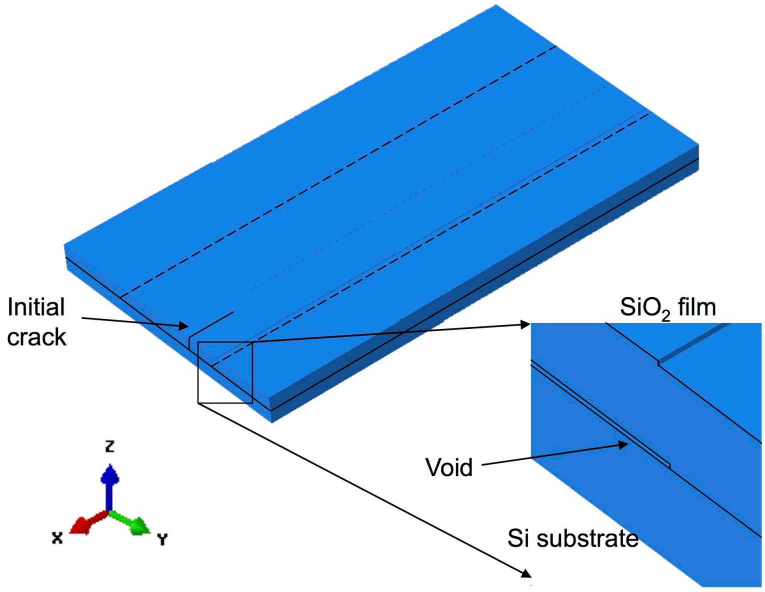

Fig. S6. 3D modeling of 20-μm wide, 100-nm high void sample.

Section 2. Stress source of e-beam exposure case

2-1. Heating level estimation

According to diffusion theory, the time needed for heat to diffuse through 2-μm-thick SiO_2 would be $t = (2\ \mu m)^2 / \alpha \approx 4.8\ \mu s$, where the thermal diffusivity of oxide is $\alpha = 0.84\ \mu m^2 / \mu s$ (1). Silicon heat diffusion time is negligible compared to that of oxide because the diffusivity of silicon is 2 orders of magnitude higher. In the experiment, the scanning time was a few seconds; thus, we could conclude that the thermal state of the sample surface is a steady state. If we assume that all the energy was focused on the probe point without scanning and the electron beam encountered the bulk SiO_2 specimen instead of the thin film system, then we could write the (overestimated) expected temperature rise as $\Delta T = 3V_{acc}I / (2\pi\kappa R)$ (2) , where probing current is $I = 1$ nA, thermal conductivity of SiO_2 is $\kappa = 1.38$ W / m ·K, and electron range R of SiO_2

is ~3 μm at V_{acc} = 20 kV, which is much larger than the probe diameter (on the order of ~100 nm),

$$\Delta T = 3V_{acc}I / (2\pi\kappa R) = (3 \times 20 \text{ kV} \times 1 \text{ nA}) / (2\pi \times 1.38 \text{ Wm}^{-1}\text{K}^{-1} \times 3 \text{ μm}) = 2.31 \text{ K} .$$

This result should be lower if we take into account the scanning of the probe beam and thermal conductivity of the silicon substrate. This result is also consistent with earlier reports of 70 K on the SiO_2 film on the Si substrate, with much higher current (600 nA) level and much smaller exposed area (2 x 2 μm^2) (1).

2-2. Estimated thermal stress

When the thickness of the film (2 μm) is much thinner than that of the substrate (500 μm) and this system is exposed to a temperature change ΔT, then the interior of the film away from the edge is subjected to the equi-biaxial in-plane stress change, $\Delta\sigma = E\Delta\alpha\Delta T / (1 - \nu)$ where E: Young's modulus; $\Delta\alpha$: difference of thermal expansion between that of film and substrate; ν: Poisson ratio of film (3). Because the thermal expansion of Si is much higher than that of SiO_2, the film is subjected to tensile stress. For the PMMA interlayer sample, we could observe crack generation from ~ 300 °C. If we take into account the material properties of SiO_2 and Si, then this enhanced tensile stress at 300 °C is

$$\Delta\sigma = E\Delta\alpha\Delta T / (1 - \nu) = (70 \text{ GPa})[(2.6 - 0.56) \times 10^{-6} \text{ K}^{-1}](300 - 25) \text{ K} / (1 - 0.17) = 47.3 \text{ MPa} .$$

2-3. Electrostatic charging

If electrostatic charging-induced stress is at the 50 MPa level, then we could say that the electrostatic force could drive crack growth. When the surface potential of the e-beam-exposed SiO_2 is U_s, then the initial FIB-milled initiation notch could be interpreted as a parallel capacitor plate with a 200-nm gap. Then, the electrostatic force per unit area is

$$F / A = \sigma_e = Q \times Q / (2A\varepsilon_0) \times 1 / A = (\varepsilon_0 A U_s^2) / (2d^2) \times 1 / A = (\varepsilon_0 U_s^2) / (2d^2) .$$

Assuming that U_s is x volts, then the electrostatically induced stress is

$$\sigma_e = (8.85 \times 10^{-12} \text{ C}^2\text{N}^{-1}\text{m}^{-2}) (x^2\text{V}^2) / [2(2 \times 10^{-7}\text{m})^2] = 110.625 \times x^2 \text{ Pa} .$$

Thus, if U_s is at the ~ 650 V level, then the charging-induced stress is sufficient for crack growth.

Movie S1. Real-time recording of e-beam exposure-induced crack growth. Crack is stopped by discontinuity (dot-like) of the PMMA pattern at the end of the movie.

Movie S2. Real-time dark-field recording of a metal crack sample (spiral) with a droplet of DI water. The color change of the crack gap indicates that water moves

within the crack pattern via the combined effect of capillary force and the evaporation of water.

References

1. Chu, D., Bilir, D. T., Pease, R. F. W. & Goodson, K. E. Submicron thermocouple measurements of electron-beam resist heating. *J. Vac. Sci. Technol. B* **20,** 3044 (2002).
2. Reimer, L. *Scanning electron microscopy: physics of image formation and microanalysis; 2nd ed.* (Springer, 1998).
3. Hutchinson, J. W. *Stresses and failure modes in thin films and multilayers, Notes for a DCAMM course, Technical University of Denmark.* 1–45 (published through Harvard University, 1996).
